# Letters to the Editor

**Published:** 1986-04

**Authors:** R. R. M. Harman


### Letters to the Editor

Sir,

*H.G.M. (January 1985, Volume 100, Page 22)*

H.G.M. points to the huge cost of costing the expenditure of seven clinicians for a year in a single management budgeting trial (£25,000) and also comments on the '9 til 5 Monday to Friday hours which our administrators work'. Some do, some don't. For example. a further costly study (£40,000 we are told) of the administrative and clerical costs of our local centre of excellence with the help of the Management Services Branch of the D.H.S.S. has been under way recently. Our own Department has depended for its survival on unpaid secretarial overtime for many years and I saw a chink of light as the Management Services Branch Rep. came to inspect our secretaries every fifteen minutes during the survey period to see just what they did. Could it be that at last Management would have accurate, independent information and be forced finally to recognise the load carried and hours worked? Alas, the inspecting D.H.S.S. Rep. went off duty sharp at 5 p.m. while we went on as usual another 1½ hours or so. Our secretarial staff start at 8.30 a.m. too. A request to see the Rep's superior in or out of hours was not met.

What was the quality and accuracy of that particular clinical study? Will the money be well-spent? Quis custodiet custodes?

R. R. M. Harman

